# Risk of intracranial bleeding in patients with primary brain cancer receiving therapeutic anticoagulation for venous thromboembolism: A meta‐analysis

**DOI:** 10.1002/brb3.1638

**Published:** 2020-04-21

**Authors:** Angelo Porfidia, Marzia Giordano, Carmelo L. Sturiale, Sonia D’Arrigo, Marco P. Donadini, Alessandro Olivi, Walter Ageno, Roberto Pola

**Affiliations:** ^1^ Department of Medicine Fondazione Policlinico Universitario A. Gemelli IRCCS Rome Italy; ^2^ Department of Medicine Fondazione Policlinico Universitario A. Gemelli IRCCS Università Cattolica del Sacro Cuore Rome Italy; ^3^ Department of Neurosurgery Fondazione Policlinico Universitario A. Gemelli IRCCS Rome Italy; ^4^ Department of Anesthesiology and Intensive Care Fondazione Policlinico Universitario A. Gemelli IRCCS Rome Italy; ^5^ Department of Medicine and Surgery Center on Thromboembolic Disorders and Antithrombotic Therapies University of Insubria Circolo e Fondazione Macchi ASST Sette Laghi Varese Italy; ^6^ Department of Neurosurgery Fondazione Policlinico Universitario A. Gemelli IRCCS Università Cattolica del Sacro Cuore Rome Italy

**Keywords:** anticoagulation, brain cancer, intracranial hemorrhage, meta‐analysis, venous thromboembolism

## Abstract

**Introduction:**

Venous thromboembolism (VTE) is common in glioma patients. Also, spontaneous intracerebral hemorrhage (ICH) is frequently observed in subjects with primary brain tumors. Thus, the management of anticoagulant therapy for VTE is challenging and controversial in these patients. We performed a meta‐analysis to clarify the risk of ICH in glioma patients treated with anticoagulant therapy for VTE compared to glioma patients without VTE.

**Materials and Methods:**

A systematic search of the literature was conducted using PubMed, Scopus, and EMBASE databases between January 1980 and January 2019 without language restrictions. Summary statistics for ICH were obtained by calculating the odds ratio (OR) using a random effects model, and heterogeneity across studies was estimated by the I^2^ statistic. The Newcastle–Ottawa Scale was used to evaluate the quality of studies.

**Results:**

A total of 368 studies were initially identified. Of these, 346 were excluded after title review. The remaining 22 studies were reviewed in detail. According to the PICO criteria, 15 studies were excluded. Finally, 7 studies were included in the meta‐analysis. The OR for ICH in glioma patients receiving therapeutic anticoagulation for VTE versus those who did not receive anticoagulation was 3.66 (95% confidence interval [CI], 1.84–7.29; *I* ^2^ = 31%).

**Conclusions:**

This meta‐analysis demonstrates that anticoagulation for VTE increases the risk of ICH in subjects with malignant brain tumors. Future studies are warranted to fully understand the best medical treatment of VTE in glioma patients.

## Graphical abstract

Venous thromboembolism (VTE) is common in glioma patients. Management of anticoagulant therapy for VTE is challenging and controversial in these patients. Anticoagulation for VTE increases the risk of ICH in subjects with malignant brain tumors.

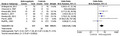

## 1 INTRODUCTION

Venous thromboembolism (VTE) is common in malignant glioma patients. Data suggest that the annual risk of deep vein thrombosis in these patients can be as high as 18% with a cumulative lifetime risk of approximately 30% (Brandes et al., 1997; Drappatz, Schiff, Kesari, Norden, & Wen, 2007). Also, spontaneous intracerebral hemorrhage (ICH) is frequently observed in subjects with primary brain tumors (Wakai, Yamakawa, Manaka, & Takakura, 1982). Thus, in these patients, the management of anticoagulant therapy for both prevention and treatment of VTE is complex and challenging (Jo, Schiff, & Perry, 2014; Perry et al., 2010; Porfidia, Morretti, & Landolfi, 2014; Senders et al., 2018). Few data are available on the risk of ICH in malignant glioma patients who use anticoagulants for the treatment of VTE.

We performed a systematic review and meta‐analysis of the studies that have evaluated the occurrence of ICH in subjects with malignant primary neoplasms of the brain, who were diagnosed with VTE and, for this reason, were treated with full‐dose anticoagulant therapy compared with malignant glioma patients without VTE not taking anticoagulant therapy.

## 2 METHODS

Data reporting in this review are consistent with the Preferred Reporting Items for Systematic Reviews and Meta‐Analyses statement (Moher, Liberati, Tetzlaff, & Altman, 2009). The review questions were formulated following the PICO criteria (Population, Intervention, Comparator, and Outcome). A systematic search of the literature was conducted using PubMed, Scopus, and EMBASE databases between January 1980 and January 2019 without language restrictions. The search strategy used a combination of the following keywords: glioma, glioblastoma, oligodendroglioma, astrocytoma, oligoastrocytoma, anticoagulant, heparin, low‐molecular‐weight heparin, vitamin k antagonist, direct oral anticoagulant (DOAC), and new oral anticoagulant.

The inclusion criteria were as follows: case–control or cohort studies or randomized trials that enrolled patients with primary malignancy of the central nervous system; a study group treated with therapeutic doses of anticoagulants (including warfarin, low‐molecular‐weight heparin or unfractionated heparin, DOACs) for VTE and a control group without VTE not treated with anticoagulants; and available information on the occurrence of intracranial bleeding in both groups.

The list of potentially eligible studies was reviewed by two independent reviewers (A.P. and M.G.). The reference lists of retrieved articles were also scrutinized to identify other publications of interest that were missed in the first search (forward search). Disagreements between reviewers were resolved by consensus.

Data extraction was performed by two authors using a standardized form. Extracted information included the following: study design, sample size, baseline population characteristic, type of anticoagulant, and incidence of ICH.

The primary analysis was conducted on the rate of ICH in patients affected by brain cancers receiving full‐dose anticoagulants for VTE compared with those not receiving anticoagulation.

The Newcastle–Ottawa Scale (NOS) was used to evaluate the quality of cohort studies and case–control studies, acknowledging that a standardized quality rating for cohort studies is lacking. The NOS evaluates the selection of cohorts (4 criteria), comparability of cohorts (1 criterion) and assessment of outcomes (3 criteria) and, for case–control studies, the selection of cases and control (4 criteria), comparability of cases and controls (1 criterion), and assessment of exposure (3 criteria) (Wells et al., 2009).

Institutional review board approval was not required in our Institution for a systematic review and a meta‐analysis.

### 2.1 Statistical analysis

Data of the study populations were reported as per individual studies. The number of patients who experienced and did not experience ICH was extracted both in the anticoagulant and in the control group. A random effect model using inverse variance weighting was used to summarize the data. The heterogeneity of pooled data was estimated by calculating the Q and *I* ^2^ statistics, and it was regarded as significant when *p* < .05 or *I* ^2 ^≥50%. Review Manager version 5.3 (Nordic Cochrane Centre, The Cochrane Collaboration, Copenhagen 2014) was used for pooling data.

## 3 RESULTS

### 3.1 Study selection

**FIGURE 1:**
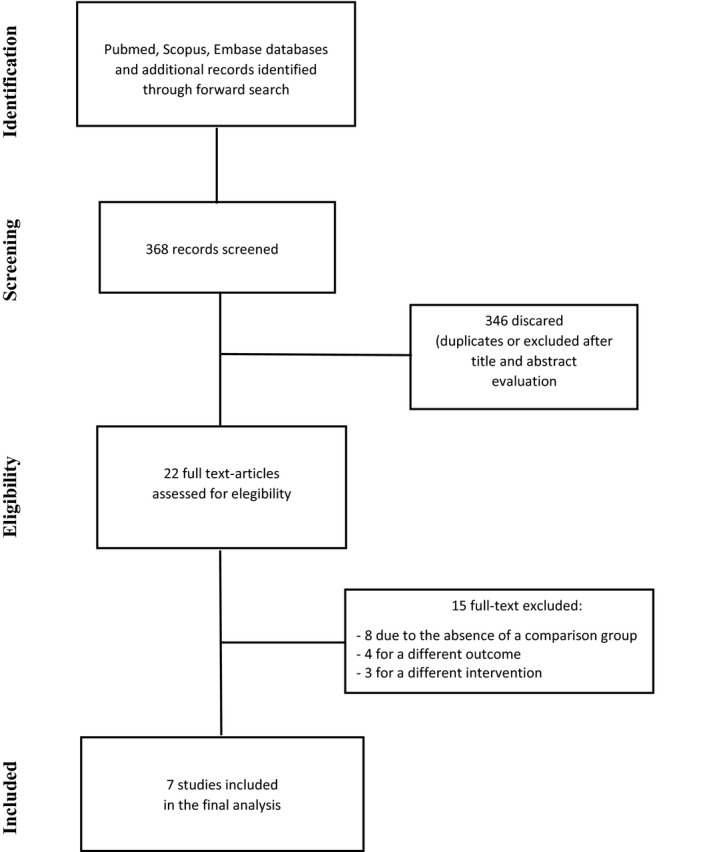
Study flow diagram

A total of 368 studies were initially identified. Of these, 346 were excluded after title review because they were not relevant for the purpose of the study. The remaining 22 studies were reviewed in detail. According to the PICO criteria, 15 studies were excluded. In particular, eight studies were excluded because no comparison group was available (Altschuler, Moosa, Selker, & Vertosick, 1990; Carney et al., 2019; Chaichana et al., 2013; Nghiemphu, Green, & Pope, 2008; Quevedo, Buckner, Schmidt, Dinapoli, & O'Fallon, 1994; Robins et al., 2008; Schmidt, Faul, Dichgans, & Weller, 2012; Simanek, Vormittag, & Hassler, 2007), three studies were excluded for a different intervention (Perry et al., 2009, 2010; Senders et al., 2018) (prophylactic doses of anticoagulant drugs), and four studies were excluded because the outcome ICH was not available (Brandes et al., 1997; Chang et al., 2005; Edwin et al., 2016; Zincircioglu et al., 2012). Finally, 7 studies were included in the meta‐analysis (Al Megren, De Wit, Al Qahtani, Le Gal, & Carrier, 2017; Choucair, Silver, & Levin, 1987; Khoury et al., 2016; Mantia et al., 2017; Norden et al., 2012; Pan, Tsai, & Mitchell, 2009; Ruff & Posner, 1983) (Figure 1).

### 3.2 Study characteristics

The characteristics of studies included in the meta‐analysis are shown in Table 1. All the included studies were retrospective (5 cohort studies and 2 case–control studies), and the indication for anticoagulant use was always acute VTE.

**TABLE 1:** Characteristics of studies included in the meta‐analysis

Author (year)	Country	Study design	Enrolment period	No of patients	No on AC	No without AC	Type of AC	Total No of ICH	No of ICH in AC group	No of ICH in control group	VTE in both groups
Mantia et al. (2017)	United States	Retrospective	2000–2016	133	50	83	LMWH	25	14	11	No
Pan et al. (2009)	United States	Retrospective	2001–2005	146	25	121	LMWH VKA	3	3	0	No
Choucair et al. (1987)	United States	Retrospective	1977–1986	36	22	14	UFH VKA	0	0	0	Yes
Ruff and Posner (1983)	United States	Retrospective	1977–1980	375	103	272	UFH VKA	8	2	6	No
Norden et al. (2012)	United States	Retrospective	NA	282	64	218	LMWH VKA	14	7	7	No
Khoury et al. (2016	United States	Retrospective	2007–2013	173	97	76	LMWH UFH VKA	17	15	2	Yes
Al Megren et al. (2017)	Canada	Retrospective	2010–2015	146	70	76	LMWH UFH Fondaparinux Unknown AC	13	11	2	No

Abbreviations: AC, anticoagulation; ICH, intracranial hemorrhage; LMWH, low‐molecular‐weight heparin; UFH, unfractionated heparin; USA, United States of America; VKA, vitamin K antagonist; VTE, venous thromboembolism.

### 3.3 Quality assessment

Overall, the quality of the included studies was considered low‐to‐moderate. The risk of bias mainly deals with the comparability of cohorts and the outcome assessment in cohort studies (Table 2).

**TABLE 2:** Study quality assessment (Newcastle–Ottawa Scale)

Author (year	Cohort studies		
	Selection	Comparability	Outcome
Ruff and Posner (1983)	★★★	/	★
Choucair et al. (1987)	★★★	/	★
Pan et al. (2009)	★★★	/	★★
Norden et al. (2012)	★★★	/	★
Khoury et al. (2016)	★★★	★★	★★

	Case–control studies		
	Selection	Comparability	Exposure
Mantia et al. (2017)	★★★	★★	★★★
Al Megren et al. (2017)	★★★	★★	★★★

### 3.4 Risk of ICH

**FIGURE 2:**
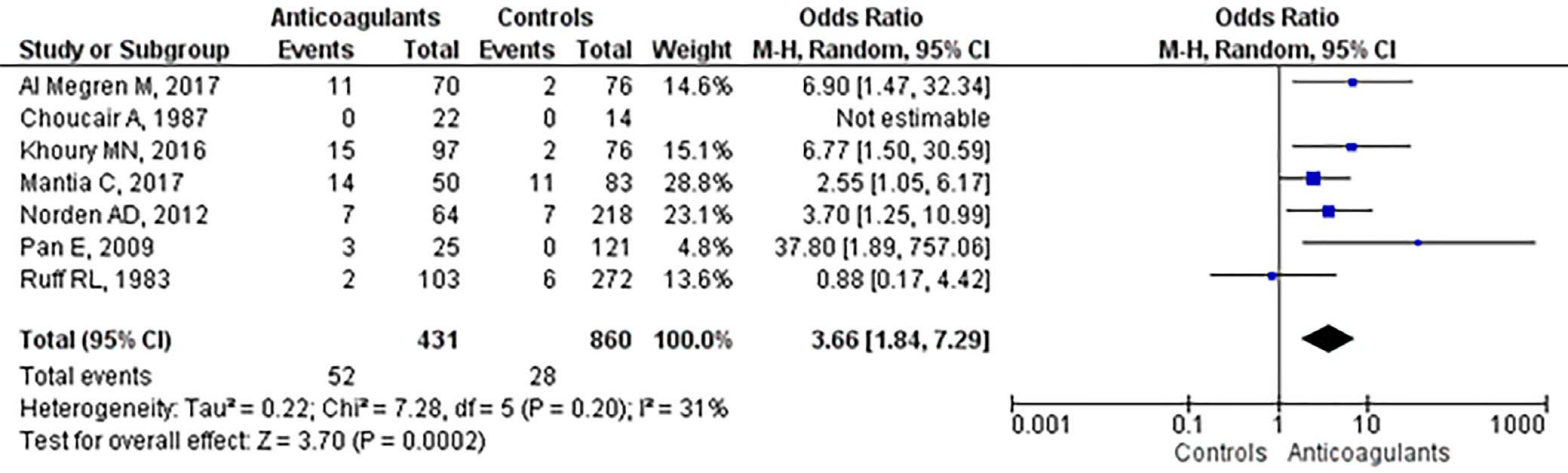
Forest plot and pooled estimate of odds ratio (OR) of intracranial hemorrhage in glioma patients receiving therapeutic anticoagulation for VTE. CI, confidence interval; VTE, venous thromboembolism

**FIGURE 3:**
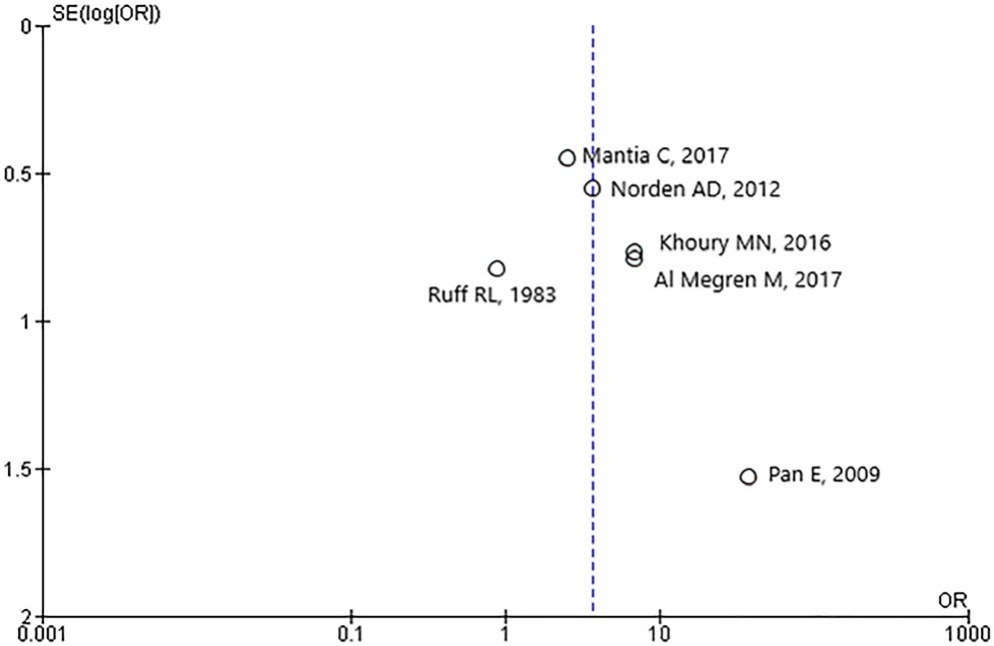
Funnel plot of standard error (SE) of studies included in the meta‐analysis. OR, odds ratio

In a random effects model, the pooled odds ratio for ICH in patients with a primary malignant brain cancer receiving therapeutic anticoagulation for VTE versus those without anticoagulation was 3.66 (95% confidence interval [CI], 1.84–7.29; *I* ^2^ = 31%) (Figure 2). In the study by Choucair and coll., odds ratio (OR) calculation was not possible because there were no reported cases of ICH (Choucair et al., 1987). Publication bias was evaluated by Funnel plot, and asymmetry of rates of hemorrhage was not apparent (Figure 3). Due to limited number of studies included in the meta‐analyses, no additional statistical analysis of bias was performed.

## 4 DISCUSSION

The results of this meta‐analysis show a significant 3.66‐fold increased risk of ICH in glioma patients treated with full‐dose anticoagulants for acute VTE as compared to patients without VTE not receiving such therapy.

In certain clinical conditions, the decision to start or withhold anticoagulant therapy for VTE is difficult. Subjects with primary malignant brain tumors who develop VTE are among those for whom the assessment of the risk–benefit ratio of anticoagulation is particularly complex, given the lack of solid scientific data to drive the best medical management. A previous meta‐analysis, which included patients with both cerebral metastases and primary malignant brain tumors, found that anticoagulant treatment for VTE was not associated with increased rate of ICH in patients with cerebral metastases, but was associated with a threefold increase in the risk of ICH in subjects with primary brain neoplasms (Zwicker, Karp Leaf, & Carrier, 2016). After this meta‐analysis, other two studies have been published and are included in the present meta‐analysis. Ours is the first meta‐analysis that is specifically limited to patients with primary malignant brain cancer.

The risk of ICH is different between the various studies included in our meta‐analysis. For instance, in the study by Al Megren et al. (2017), among 70 glioma patients treated with full‐dose anticoagulant therapy, there were 11 ICH (15.7%), compared to 2 ICH (2.4%) in glioma patients who were not taking anticoagulants. In this study, the OR for ICH in treated versus untreated patients was 7.5. On the other hand, in the study by Mantia and coll., the authors have distinguished between ICHs of different severity, from trace radiologic evidence of blood products to overt hemorrhage with mass effect (Mantia et al., 2017). In this study, any bleeding that occurred within 4 weeks after surgery was excluded from analysis. ICHs that measured more than 1 ml were classified as measurable, while hemorrhages that were more than 10 ml in volume, required surgical intervention, or were associated with clinical symptoms were defined as major. When all hemorrhages were considered as a whole, their 1‐year incidence was 28.1% in subjects receiving anticoagulant therapy and 13.6% in controls. When only measurable ICHs were considered, their 1‐year incidence was 18.8% in the anticoagulation group and 7.8% in the control group. Finally, when only major ICHs were taken into consideration, the 1‐year incidence was 14.7% in subjects receiving anticoagulation versus 2.5% in subjects that were not on anticoagulant therapy (HR 3.37). The study of Pan and coll. included subjects who have had recent surgery (Pan et al., 2009). Of 146 subjects, 41 (28%) developed VTE, 25 (17%) where treated with full‐dose anticoagulant and 3 (2%) developed ICH. Norden and coll. studied brain cancer patients treated with bevacizumab and receiving anticoagulant therapy for VTE (Norden et al., 2012) versus a control group of non‐VTE patients treated with bevacizumab alone. Bevacizumab is a humanized monoclonal antibody direct to vascular endothelial growth factor approved for the treatment of glioblastoma. The use of this drug has been associated both with an increased risk of VTE and bleeding. The authors showed a total of 14 cerebral hemorrhages, respectively, 7 in patients treated with anticoagulants and 7 in untreated patients. In this study, data regarding brain surgery are not available.

Some studies report that the median survival is similar between glioma patients with VTE who develop and do not develop ICH (Al Megren et al., 2017; Khoury et al., 2016). In another study, the median survival was similar among patients receiving and not receiving anticoagulation, although the diagnosis of ICH while receiving anticoagulants was associated with a significantly shorter survival (Mantia et al., 2017).

A potential treatment, that is alternative to anticoagulation in patients with a brain tumor and VTE, is the placement of an inferior vena cava (IVC) filter. However, this procedure has been associated with a high rate of complications including malposition, thrombosis, and pulmonary embolism (Zwicker et al., 2016). In one study, about 60% of patients with brain tumors treated with IVC filter developed complications related to the filter, including a high rate of pulmonary embolism and thrombosis of the filter (45%) (Levin et al., 1993). In a recent study, in patients with glioma and VTE who underwent placement of an IVC filter, the rate of recurrent VTE was 30% with an additional 5% of mechanical complications regardless of the presence or absence of anticoagulant treatment (Edwin et al., 2016). Thus, the use of IVC filters should be carefully evaluated for each individual patient, always taking into consideration that, for primary brain tumors, the American Society of Clinical Oncology recommends anticoagulation and does not support the routine use of IVC filter in glioma patient who develop VTE 7 (Lyman et al., 2013).

Since DOACs have shown a significant reduction in the risk of intracranial bleeding compared with vitamin K antagonists both in patients with atrial fibrillation and VTE (Van Es, Coppens, Schulman, Middeldorp, & Büller, 2014), the idea that the same advantage could be present also in subjects with brain tumors is fascinating. In one study on 67 subjects with primary brain tumor and VTE, the cumulative incidence of any ICH was 0% in patients receiving DOACs versus 36.8% in those treated with LMWH with a major ICH incidence of 18.2% (Carney et al., 2019). Based on this pivotal study, future investigation is warranted to establish the safety profile of DOACs in subjects with malignant brain tumors.

This study has some limitations. First, it is based on studies that only have a retrospective design. Second, all patients included in the studies had malignant brain tumors, but the information about nature, size, stage, and management of the cancer (surgery, chemotherapy, radiation therapy) and additional risk factors for both VTE and ICH are incomplete or absent across the studies. In addition, in the analyzed studies patients with VTE were subjected to different types of anticoagulants and heterogeneous therapeutic regimens. Finally, the studies included in the meta‐analysis did not have a protocol‐scheduled imaging for the surveillance of ICH, lacked a homogenous definition of ICH.

## 5 CONCLUSIONS

In summary, our meta‐analysis suggests that anticoagulation increases the risk of ICH in subjects with malignant brain tumors and VTE, although in some studies ICH does not influence overall survival. In the studies included in this meta‐analysis, anticoagulation was always based on the use of heparin and/or vitamin K antagonists. Future studies with DOACs might have an impact on these findings.

## CONFLICT OF INTEREST

The authors declare no financial or other conflict of interests.

## AUTHOR CONTRIBUTION

AP and CLS contributed to the concept and design of the study, drafting, and revision of the final article; AP and MG contributed to acquisition of data; CLS, SD, MPD, and WA contributed in data analysis and interpretation; RP contributed in data interpretation and drafting the paper; RP and AO supervised the project and revised the paper.

## Data Availability

The data supporting the results of this study are publicly available in literature or from the corresponding author upon reasonable request.
